# Low Fermentable Oligo- Di- and Mono-Saccharides and Polyols (FODMAPs) or Gluten Free Diet: What Is Best for Irritable Bowel Syndrome?

**DOI:** 10.3390/nu12113368

**Published:** 2020-11-01

**Authors:** Massimo Bellini, Sara Tonarelli, Maria Gloria Mumolo, Francesco Bronzini, Andrea Pancetti, Lorenzo Bertani, Francesco Costa, Angelo Ricchiuti, Nicola de Bortoli, Santino Marchi, Alessandra Rossi

**Affiliations:** 1 Gastrointestinal Unit–Department of Translational Sciences and New Technologies in Medicine and Surgery, University of Pisa, 56124 Pisa, Italy; massimo.bellini@med.unipi.it (M.B.); g.mumolo@int.med.unipi.it (M.G.M.); bronzinifrancesco@yahoo.it (F.B.); pancio10@alice.it (A.P.); lorenzobertani@gmail.com (L.B.); fcosta@med.unipi.it (F.C.); a.ricchiuti@int.med.unipi.it (A.R.); nicola.debortoli@unipi.it (N.d.B.); santino.marchi@unipi.it (S.M.); 2 Clinical and Experimental Medicine–Rheumatology Unit, University of Pisa, 56100 Pisa, Italy; alessandra.rossi@unipi.it

**Keywords:** irritable bowel disease, FODMAP, low FODMAP diet, gluten free diet, non-celiac gluten wheat sensitivity

## Abstract

Irritable Bowel Syndrome (IBS) is a very common functional gastrointestinal disease. Its pathogenesis is multifactorial and not yet clearly defined, and hence, its therapy mainly relies on symptomatic treatments. Changes in lifestyle and dietary behavior are usually the first step, but unfortunately, there is little high-quality scientific evidence regarding a dietary approach. This is due to the difficulty in setting up randomized double-blind controlled trials which objectively evaluate efficacy without the risk of a placebo effect. However, a Low Fermentable Oligo-, Di- and Mono-saccharides And Polyols (FODMAP) Diet (LFD) and Gluten Free Diet (GFD) are among the most frequently suggested diets. This paper aims to evaluate their possible role in IBS management. A GFD is less restrictive and easier to implement in everyday life and can be suggested for patients who clearly recognize gluten as a trigger of their symptoms. An LFD, being more restrictive and less easy to learn and to follow, needs the close supervision of a skilled nutritionist and should be reserved for patients who recognize that the trigger of their symptoms is not, or not only, gluten. Even if the evidence is of very low-quality for both diets, the LFD is the most effective among the dietary interventions suggested for treating IBS, and it is included in the most updated guidelines.

## 1. Introduction

Irritable Bowel Syndrome (IBS) is one of the most common gastrointestinal disorders. Patients with IBS do not have identifiable structural or biochemical abnormalities and the diagnosis is based on the Rome IV criteria, which stress the importance of abdominal pain related to defecation and change in bowel frequency and stool consistency/form [1,2].

On the basis of the consistency/form of stools, IBS patients can be subdivided into three categories:

- IBS with predominant diarrhea (IBS-D): >25% of bowel movements with Bristol stool form types 6–7;
- IBS with predominant constipation (IBS-C): >25% of bowel movements with Bristol stool form types 1–2
- IBS with mixed bowel habits (IBS-M): >25% of bowel movements with Bristol stool form types 1 or 2 and >25% of bowel movements with Bristol stool form types 6 or 7.

If the patient cannot be categorized into one of the above three categories, he/she is defined as unclassified (IBS-U) [3].

IBS has a heterogeneous and incompletely understood pathophysiology, including altered brain-gut interactions, changes of microbiome, visceral or central hypersensitivity, abnormal gastrointestinal motility, psychosocial factors and food hypersensitivity [4]. Therefore, it is not surprising that there is no standardized and universally agreed therapy for this disorder.

However, the influence of dietary triggers on the generation of IBS symptoms has always been widely recognized [5,6]. Simren and Böhn showed that a very high percentage of IBS patients correlate their symptoms with food ingestion [7,8]. In these case studies, up to 63% and 84% of IBS patients respectively, especially women, reported food-related symptoms. Bloating and abdominal pain were the most frequently reported symptoms. Carbohydrates, fatty foods, coffee, alcohol and hot spices were the most frequently reported triggers.

The mechanisms by which food can cause symptoms in IBS patients are numerous. These include immune activation and allergy, mast cell degranulation and inflammation, luminal distension, bioactive molecules, such as peptides/amines, present in food and acting by regulating gastrointestinal motility and visceral sensitivity [9,10]. Recently, the role of Fermentable Oligo-, Di- and Mono-saccharides And Polyols (FODMAPs) have been highlighted as possible mechanisms by which food could cause symptoms in predisposed patients. These act by increasing the fluid content of the intestinal lumen due to the recall of water induced by osmotic activity, forcing water into the gastrointestinal tract and increasing the production of gas by the gut microbiota as a consequence of food fermentation [11,12].

Based on the potential role of food in symptom generation, a common therapeutic approach chosen by gastroenterologists for their IBS patients is often based on lifestyle and dietary behavior suggestions [13,14].

In recent years, many different dietary approaches have been suggested for IBS symptom improvements, such as the Low FODMAP Diet (LFD), the Gluten Free Diet (GFD), the wheat-free diet, the lactose-free diet and the NICE (National Institute for Health and Care Excellence) diet. In addition, many different do-it-yourself diets are also very frequently followed by patients, with non-scientifically motivated restrictions of one or more categories of food. These are often suggested by friends and relatives, the media and/or star system celebrities and imply a high risk of nutritional inadequacy [15].

Unfortunately, as highlighted by Dionne et al., the evidence concerning the efficacy of the different diets in IBS is often of a low quality [16].

The main limitations of clinical trials regarding the dietary therapy for IBS are:

- The difficulty in establishing an effective blinding. This is because over the years IBS patients continue or simply come to know many diets commonly suggested for IBS therapy. This makes it difficult to create a blind trial as the patients often recognize these different diets when they are suggested to the patients.
- The unclear adherence rates, except for very expensive and complex studies, such as trials that provide patients with all the food needed for the study.
- The unclear evidence about the right length of wash out period in crossover studies in order to avoid carry over effects on symptoms and also on gut microbiota.
- IBS dietary trials are rarely supported by pharmaceutical companies or investors as IBS is not seen as a profitable business.

For these reasons, IBS dietary studies are very different from one other and often include a limited number of patients. Therefore, most studies do not meet the GRADE guidelines level for high quality evidence [17].

The aim of this paper is to discuss the evidence regarding two of the most advised diets for IBS, the LFD and the GFD, in order to evaluate which of the two could be more suitable for IBS patients.

## 2. Gluten Free Diet

Gluten refers to a family of proteins known as prolamins (glutenin and gliadin), which are storage proteins in the starchy endosperm of many cereal grains such as wheat, barley and rye [18]. In a GFD, these cereals, and also their hybrids or derived cereals such as kamut, spelt and triticale, are not allowed. Oats are tolerated when not contaminated (however, it is necessary to check product by product). Alternatives to cereals containing gluten are rice, corn, potatoes and minor cereals or pseudocereals such as teff, millet, buckwheat, quinoa and amaranth [19]. The GFD therefore consists of a complete elimination from the diet of products containing wheat, barley and rye. This is not always simple because gluten contamination may be present in unsuspected products such as soy sauce, packet broth and malt by-products [20,21]. Table 1 reports foods that are allowed and forbidden in a GFD.

A GFD is the only recognized therapy for Celiac Disease (CD), which is an autoimmune condition characterized by a specific serological and histological profile and triggered by gluten ingestion in genetically predisposed individuals [22]. In recent years, GFD has been suggested as a possible therapy in IBS, or at least in a subgroup of IBS patients [23] (Table 2).

However, it seems important to take note of the fact that the GFD is often chosen as a “trendy diet”, also by non-celiac subjects, becoming a business worth 15 million dollars in the USA only in 2016 [34,35]. One of the reasons why the GFD is so popular is because it is considered, falsely, a “miraculous” diet, improving mental and physical performances also in healthy people. On the contrary, several studies have shown that fat intake is higher than recommended and the mean intake of protein in CD patients on a GFD is lower, as well as the consumption of vegetable proteins and dietary fibers. Moreover, a higher intake of sugars and a lower intake of calcium and vitamin B12, folate and vitamin D have been reported more frequently in CD patients on a GFD than in controls [36].

Furthermore, as well as other restrictive diets, the GFD could prompt or reinforce an eating disorder [11].

## 3. Low FODMAP Diet

FODMAPs are a large class of small non-digestible carbohydrates containing only 1–10 sugars poorly absorbed in the small bowel. FODMAPs are common in a wide range of fruit, vegetables, cereals, milk and dairy products, legumes and sweeteners.

These molecules, found undigested in the intestinal lumen, act in different ways:

- By increasing the small bowel water content as they are osmotically active;
- By increasing the production of gas through bacterial fermentation;
- By increasing the production of bacterial metabolites such as Short-Chain Fatty Acids (SCFAs).

Within the context of visceral hypersensitivity typical of IBS patients, FODMAPs may provoke abdominal pain, bloating, flatulence and bowel habit alterations [11].

Table 3 reports allowed and forbidden foods in an LFD.

An LFD consists of a first phase of global elimination of all these molecules, lasting from 4 to 8 weeks, and a subsequent phase of reintegration of one category of these carbohydrates step by step. This allows the patient, who has to be followed by a skilled nutritionist, to identify the kind and the amount of foods to which he/she is sensitive, and to find adequate alternatives. This approach enables the medical practitioner to tailor the diet to the single patient. It also ensures implementation of the diet in the long term, establishing an Adapted Low FODMAP Diet (AdLFD), thus minimizing the risks of possible nutritional inadequacy [37].

The involvement of a skilled nutritionist is mandatory. This is because the reliability of information reported by patients regarding the “trigger” FODMAP-containing foods can be questionable, even in a gastroenterological setting. Indeed, Bellini et al., comparing what the patient thought before starting the LFD and the intolerance detected by the nutritionist after the reintroduction phase, found that patients’ reliability in detecting the real FODMAP provoking their symptoms is generally poor or fair [38].

Although the evidence is of very low quality, an LFD had the greatest efficacy among dietary interventions suggested for treating IBS symptoms [16].

A meta-analysis by Marsh showed a significant decrease in the IBS-SSS (IBS Severity Scoring System) and an improvement in abdominal pain, bloating and IBS-QOL (IBS Quality of Life) [39].

Another meta-analysis by Schumann found that the LFD, in comparison to other diets, including the usual dietary recommendations for IBS, was effective and safe in the short term [40].

A global improvement in all parameters related to bowel habits was observed also by Bellini et al. in a group of IBS patients: the IBS-SSS global score and the scores of the single items significantly improved after the eight-week LFD [41].

However, some potential limitations and concerns of LFDs have been raised because it can

- be complex and difficult to teach and learn, because it consists of several steps and requires time, motivation and the involvement of an expert in nutritional matters;
- be potentially expensive, due to the choice of more expensive, and difficult to find alternative foods;
- reduce the normal intake of natural prebiotics, strongly modifying the gut microbiota;
- increase the risk of constipation, limiting fiber intake;
- be nutritionally inadequate;
- favor the onset of or precipitate an eating disorder behavior;
- be ineffective in the long term.

Only a few studies have assessed the long-term effects of the LFD, both in terms of efficacy and safety from a nutritional point of view [11].

In a study of our group, an eight-week LFD, monitored by a skilled nutritionist, caused no changes in energy, macronutrients or fiber intake. There were no effects on nutritional status and body composition, whereas other studies have found changes in the introduction of micro- and macronutrients during a strict LFD [41,42,43].

However, most of the studies that have evaluated nutritional adequacy have been based exclusively on the first phase of the LFD, while the second phase, the AdLFD, which is the diet that has to be undertaken in the long run, was rarely evaluated. Since no single food group is completely eliminated during the AdLFD, it is unlikely that patients would encounter a significant and dangerous nutritional imbalance.

In fact, O’Keeffe, evaluating the personalized diet in the long term, found no differences regarding energy and nutrient intakes between an habitual diet and an AdLFD, with higher levels of folate and vitamin A in the AdLFD [44].

Furthermore, Harvie reported that after a decrease in energy and fiber intake during the strict LFD, both energy and fiber increased to levels similar to those of the habitual diet during the AdLFD [45].

Very recently, Bellini et al., in a study involving 73 IBS patients, showed that the LFD was effective in controlling digestive symptoms both in the short and long term, and in improving quality of life, anxiety and depression, even if some problems regarding acceptability were reported and adherence decreased in the long term [38]. The diet also improved the food-related quality of life without affecting nutritional adequacy.

## 4. Non-Celiac Gluten/Wheat Sensitivity and IBS

A GFD is often suggested to patients with IBS-like symptoms (abdominal pain, diarrhea, bloating and flatulence). Indeed, Vazquez-Roque et al. showed that in these patients gluten caused a decrease in the expression of tight junction proteins in the colonic mucosa, causing an alteration of bowel barrier functions, especially in patients with HLA DQ8/2, the same as celiac patients [27].

**Figure 1:**
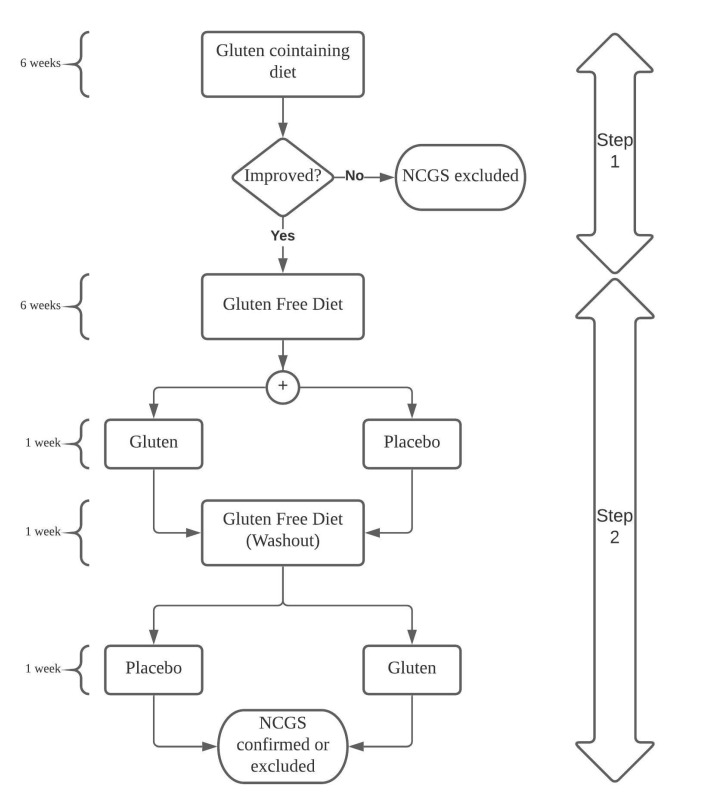
Gluten challenge scheme. NCGS: Non-Celiac Gluten Sensitivity.

In 1978, the term “Non-Celiac Gluten Sensitivity (NCGS)” was coined by Ellis and Linaker [46]. It is a clinical entity which seems often to overlap with IBS. It is characterized by gastrointestinal (GI) and extra-GI symptoms (headache, foggy mind, chronic fatigue, joint pain, tingling or numbness of the extremities, eczema) associated with gluten ingestion (occurring hours or days after the ingestion) in individuals in whom CD and wheat allergy have been excluded [47]. The diagnosis of certainty, according to the Salerno Expert consensus, is based on a close and standardized monitoring of the patient during elimination and reintroduction of gluten, in the absence of specific biomarkers [48]. This “gluten challenge” is composed of two phases (Figure 1). In the first phase patients have to maintain a gluten-containing diet for at least six weeks. After that, they start a GFD for six weeks. The second phase consists of the reintroduction of gluten (8 g of gluten per day) or placebo. For one week the patient receives the GFD and gluten or placebo, followed by a one-week washout (strict GFD) and then by the crossover with gluten or placebo for another week. In both phases the patients monitor their symptoms according to the Gastrointestinal Symptom Rating Scale (GSRS) and a Numerical Rating Scale (NRS) with a score ranging from 1 (mild) to 10 (severe). A variation in the symptom severity of at least 30% between the gluten and placebo challenge discriminates a positive from a negative result. This challenge is often performed with a single-blind approach, more suitable for clinical practice.

However, the debate is still open as to whether the gluten is the culprit [30,31,48]. Indeed, also in non-gluten free food there are other molecules potentially responsible for the symptoms such as Wheat Germ Agglutinins (WGA), which induce the release of pro-inflammatory cytokines and act on the intestinal barrier, amylase trypsin inhibitors (ATIs), pest resistance molecules and activators of innate immune responses in human and murine models. Moreover, wheat also contains fructans, which are FODMAPs [49].

In 2011, Biesiekierski et al. showed that gluten caused both GI and extra-GI symptoms in non-celiac patients [26]. However, two years later, the same group reported the results of a study demonstrating that an LFD had good results in NCGS patients who had previously responded to a GFD. The symptoms then worsened with the intake of three alternative diets (low gluten, high gluten or control), with only 16% that had a worsening of symptoms in the high gluten diet. Furthermore, only a small percentage (8%) did not fail the rechallenge with gluten [50].

Some studies report that only a small percentage of patients with self-diagnosed NCGS are truly hypersensitive to gluten. In Molina-Infante’s analysis, including a sample of 1312 patients, only 16% of the patients had gluten-specific symptoms, while 40% showed a nocebo response (similar or more severe symptoms in response to the placebo than with gluten) to the reintegration of gluten [51]. These results support the idea that the role of gluten is still unclear due to the high risk of placebo and nocebo effects [52].

Skodje et al. suggests that fructans are actually those most responsible for the symptoms of these patients. In a double-blind crossover challenge of 59 non-celiac subjects on a self-instituted gluten free diet the mean overall GSRS-IBS score (Gastrointestinal Symptom Rating Scale, Irritable Bowel Syndrome version) for participants consuming fructans was significantly higher than for those consuming gluten, as was the GSRS bloating sub-dimension [53]. There was no difference in GSRS-IBS scores between gluten and placebo groups. However, Volta et al. point out the limitations of this study: the authors did not use the Salerno Criteria for NCGS, but they simply enrolled self-diagnosed NCGS. In addition, some extra-GI symptoms typical of NCGS were not included in the evaluation, and data on the presence of ANCA (anti-neutrophil cytoplasmic antibodies) and anti-gliadin IgG were incomplete [54].

The results of the Skodje and Biesiekierski studies, taken together, suggest that also fructans and/or other components of wheat, and not only gluten, could be the culprits regarding the symptoms in NCGS patients. These could therefore be more precisely defined as “Non-Celiac Wheat Sensitive” (NCWS) [52].

In light of these data, NCWS patients could possibly be considered as a subset of IBS patients particularly sensitive to wheat [23].

Dieterich claims that 19 self-diagnosed NCGS patients’ GSRS improved during a LFD from 13.8 ± 6.2 to 8.7 ± 5.2 (*p* < 0.001), and on a GFD (4.6 ± 4.3; *p* < 0.05), but some symptoms improved more markedly with the GFD, with both abdominal pain and alterations of bowel function responding better to the GFD [55]. This result, the LFD being more restrictive and therefore potentially more effective, is counterintuitive. However, this study did not consider the possible placebo effect that the GFD could have had in the NCGS patients. Indeed, due to the lack of blinding, the patients were able to recognize the two different diets. 

**Figure 2:**
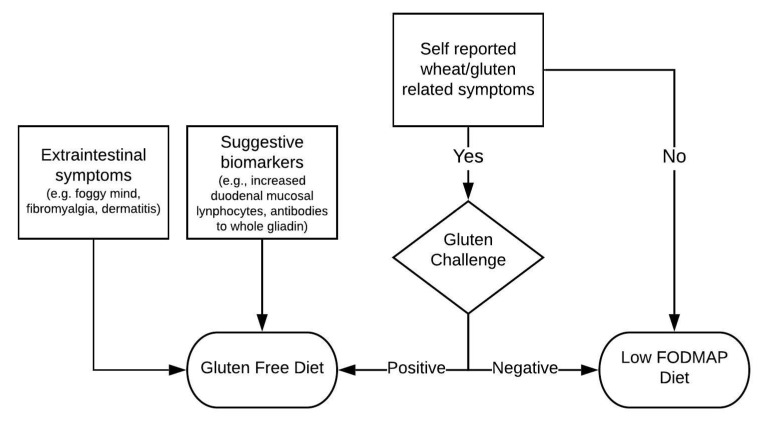
Algorithm for the choice between GFD and LFD in IBS.

It is therefore up to the clinician to understand which category each patient most likely belongs to and which diet will benefit him/her the most. This decision is fundamentally whether the patient is a NCWS patient with IBS-like symptoms responding to a GFD or is an IBS patient not sensitive to wheat or sensitive not only to wheat, for whom an LFD may be more suitable, the LFD involving a more comprehensive exclusion of potentially harmful foods. In Figure 2, a flow chart describing the management of such patients is shown. 

The application in real life of a therapeutic algorithm of this kind, which includes a double-blind gluten challenge, is obviously complicated. This makes NCWS an entity of complex identification and controversial in nature, thus being closely related to the placebo/nocebo effect.

## 5. Gluten Free Diet vs. Low FODMAP Diet

Therefore, what should be the most recommended diet therapy for IBS patients?

As mentioned by De Giorgio et al. and by Dionne et al., there is evidence reporting that an LFD is more effective in IBS patients than a GFD [9,16]. Recently, in a study by Paduano et al., the LFD was more effective than the GFD and a balanced diet in decreasing abdominal bloating. It was also the only regimen able to normalize the bowel function by reaching the Bristol stool form type 4 [32].

The nature of the LFD, consisting of a phase of monitored and tailored reintroduction of foods based on the tolerance/intolerance of the single patient, ensures better nutritional safety. It does not necessarily eliminate completely any category of food such as that implied by the GFD. It is however to be considered, in the choice of diet suitable for the individual patient, that in some cases, when many different foods are responsible for symptoms, even the AdLFD can be very restrictive in order to ensure a greater resolution of the symptoms. 

It is consequent that even during an AdLFD in wheat-sensitive patients, it may be necessary to eliminate or greatly reduce products containing wheat. This therefore involves making extensive use of gluten-free products, which are more expensive and less nutritionally adequate than the counterparts containing gluten. This is a problem that the patients can overcome only by preparing home-made products with naturally gluten-free flours and following the suggestions of a skilled nutritionist [9,41].

Although the LFD in the first phase (the elimination of all FODMAPs) may cause an increase in spending on food, the second phase, the reintroduction phase in which a more relaxed and liberal diet is allowed, can involve reduced costs [38,44].

Evaluating the effects of both diets on the gut microbiota is difficult because studies have used different designs and methods, thus making the results not always comparable, but this is another interesting matter of discussion. A GFD seems to induce a reduction in *Bifidobacteria* and *Lactobacilli*, similarly to the LFD in the first phase [43,56,57]. Thus, as the LFD is designed to be adapted to the individual patient, with the reintroduction of a range of initially forbidden foods, it probably has a lower negative influence on gut microbiota. Harvie et al. evaluated the microbiota of patients at the end of the reintroduction of FODMAP foods and found no alteration in OTUs (Operational Taxonomic Units) after dietary intervention [45].

Finally, a GFD could be useful for those patients who report extraintestinal symptoms or have biomarkers suggesting specific symptoms (i.e., increased duodenal mucosal lymphocytes in a duodenal biopsy or serum anti-gliadin antibodies). A gluten challenge could be advisable for those reporting symptoms mainly linked to gluten ingestion, and an LFD could be directly suggested to patients without wheat/gluten related symptoms (Figure 2). This is true even if, as reported above, some evidence shows how often the perception of intolerances subjectively reported by the patients are not very reliable. Furthermore, IBS patients are not always aware of the foods really able to trigger symptoms, thus making self-diagnosed gluten intolerance somewhat unreliable [38,51]. 

## 6. Conclusions 

The overlap of symptoms between NCWS and IBS, the lack of reliable markers for the diagnosis of both of them and the possibility that they could both benefit from similar types of diets generate difficulties in clearly distinguishing and characterizing this relatively new disease which deserves further studies to clarify the controversial aspects still existing. IBS patients complaining of symptoms exclusively, or mainly, linked to gluten or wheat, could benefit from a GFD as the first-line diet therapy [23]. This is because the LFD, especially in its strict phase, is a complex diet requiring close monitoring by a nutritionist expert, who is not always available. In IBS patients who report their symptoms linked to food, but not due, or not only due, to gluten/wheat ingestion, an LFD appears to be the best option. Under the careful guidance of a skilled nutritionist the LFD is nutritionally adequate and can be followed also in the long term [38]. Moreover, an LFD, with its phase of careful reintroduction of the single FODMAP categories, enables the clinician, and the patient, to have a more precise knowledge of individual sensitivity. Since the first approach to IBS patients should be based on reassurance and changes in lifestyle and dietary behavior, the LFD enables them to learn more about their own disease and about foods triggering their symptoms. However, it should be highlighted that both the GFD and LFD, as they are elimination diets, can be perceived as difficult to initiate and to continue for a lifetime by IBS patients, and consequently, their application and usefulness in daily life should be periodically and carefully monitored both by the gastroenterologist and nutritionist. 

## Figures and Tables

**Table 1:** Gluten free diet: allowed and forbidden foods.

Allowed Foods	Forbidden Foods
Corn	Wheat
Potatoes	Barley
Rice	Rye
Millet	Malt
Buckwheat	Kamut
Quinoa	Spelt
Amaranth	Triticale
Teff	Bulgur
Oats, if free from contamination	Beer Malt

**Table 2:** Studies on Gluten free Diet (GFD) in Irritable Bowel Syndrome (IBS).

	Patients	Methods	Evaluated Parameters	Results
Wahnschaffe et al. [24] 2001	IBS-D = 26	6 months GFD	Stool frequency IgA anti-gliadin IgA anti-tTG IEL count HLA DQ2	Improved stool frequency in patients with HLA DQ2.
Wahnschaffe et al. [25] 2007	IBS-D = 41	6 months GFD	Stool Frequency IBS symptoms questionnaire (Likert) HLA DQ2	Stool frequency and GI symptom score returned to normal values in 60% of IBS patients who were positive and in 12% who were negative for HLA DQ2.
Biesiekierski et al. [26] 2011	IBS = 34	6 weeks gluten or placebo containing bread with GFD	HLA DQ2/8 IBS symptoms questionnaire (VAS)	56% having HLA DQ2/8. 68% in the gluten group reported that symptoms were not adequately controlled compared with 40% on placebo.
Vazquez-Roque et al. [27] 2013	IBS-D = 45	4 weeks gluten containing diet or GFD	Bowel function Small bowel and colonic transit Lactulose and mannitol excretion HLA DQ2/8	The gluten containing diet increased bowel frequency in HLA DQ2/8 patients and was associated with higher intestinal permeability.
Aziz et al. [28] 2015	IBS-D = 41	6 weeks GFD	IBS-SSS HADS FIS SF-36 HLA DQ2/8	GFD reduced IBS-SSS by ≥50 points in 71%. HLA DQ2/8 positive subjects had a greater reduction in depression score and increase in vitality score.
Shahbazkhani et al. [29] 2015	IBS = 72	6 weeks GFD + 6 weeks gluten powder or placebo	IBS symptoms questionnaire (VAS)	Improvement was statistically different in the gluten containing group compared with placebo group in 25% and 83% patients, respectively.
Zanwar et al. [30] 2016	IBS = 60	4 weeks GFD + 4 weeks washout + 4 weeks DBPC rechallenge	IBS symptoms questionnaire (VAS)	Gluten group scored significantly higher in abdominal pain, bloating and tiredness and their symptoms worsened within 1 week of the rechallenge.
Barmeyer et al. [31] 2017	IBS-D/M = 35	4 months GFD	SGA IBS-SSS IBS-QoL EQ-5D HLA DQ2/8	HLA DQ2/8 was not associated with wheat sensitivity. 34% of the patients reported considerably or completely relieved symptoms on the GFD.
Paduano et al. [32] 2019	IBS = 42	4 weeks LFD + 4 weeks GFD + 4 weeks Mediterranean diet	Bristol stool scale IBS-SSS IBS-QoL IBS symptom questionnaire (VAS) SF-12	After GFD, improvement in symptoms, in particular, VAS bloating, VAS pain and IBS-SSS, with a smaller improvement in bloating compared to the low FODMAP diet, but with an adherence index of only 11%.
Pinto-Sanchez et al. [33] 2020	IBS = 50	4 weeks GFD	GI transit Birmingham IBS questionnaire Bristol Stool Scale HADS STAI-TAY PHQ-15 PGWB Anti-gliadin	After the GFD, patients with anti-gliadin reported less diarrhea. IBS symptoms improved in 75% of the patients with anti-gliadin and in 38% without the antibodies. GI transit normalized in a higher proportion of patients with anti-gliadin.

DBPC: Double-Blind Placebo-Controlled; EQ-5D: European Quality of Life-5 Dimensions; FIS: Fatigue Impact Scale; GFD: Gluten Free Diet; GI: Gastrointestinal; HADS: Hospital Anxiety and Depression Scale; HLA: Human Leukocyte Antigens; IBS: Irritable Bowel Syndrome; IBS-D: Irritable Bowel Syndrome Diarrhea; IBS-M: Irritable Bowel Syndrome Mixed; IBS-QoL: Irritable Bowel Syndrome Quality of Life; IBS-SSS: Irritable Bowel Syndrome Symptom Severity Score; IEL: Intraepithelial Lymphocytes; IgA: Immunoglobulin A; LFD: Low Fermentable Oligo-, Di- and Mono-saccharides And Polyols (FODMAP) Diet; PGWB: Psychological General Well-Being; PHQ-15: Patient Health Questionnaire; SF-12: Short Form 12; SF-36: Short Form 36; SGA: Subject’s Global Assessment; STAI-TAY: State-Trait Anxiety Inventory; tTG: Tissue Transglutaminase; VAS: Visual Analogue Scale.

**Table 3:** Low FODMAP diet: Allowed and forbidden foods.

Food Categories	Allowed Foods	Forbidden Foods
Cereals	Rice, porridge, oats, quinoa, tapioca, millet, amaranth, buckwheat, gluten-free bread and cereals, potato-flour.	Bread and bakery products, biscuits, croissants, pasta, wheat flour, Kamut, barley, rye, couscous, flour, muesli.
Milk and derivates	Lactose-free milk, rice milk, oat milk, soy milk and all vegetable drinks, yogurt lactose free, soy yogurt, Greek yogurt, hard cheeses, fruit sorbets.	Cow milk, goat milk, yogurt with lactose, fresh cheeses, butter, ice cream, cream.
Vegetables	Carrot, pumpkin, Chinese cabbage, celery, lettuce, spinach, potato, tomato, zucchini, eggplant, green bean, beets, red pepper, herbs, olives, bamboo shoot, fresh herbs.	Asparagus, cauliflower, garlic, onion, shallot, mushroom, leek, chicory, fennel, artichoke, Brussel sprout, broccoli, radish, pepper, turnips, Jerusalem artichoke.
Legumes	Peas, soy products.	Beans, chickpeas, lentils, soybeans.
Fruit	Banana, blueberry, strawberry, raspberry, grape, melon, grapefruit, kiwi, orange, lemon, limes, pineapple, passion fruit.	Apple, pear, watermelon, mango, apricot, avocado, cherry, peach, plum, persimmon, lychee, fruit juices.
Dried fruits	Almonds, hazelnuts, walnuts, pine nuts.	Pistachios, cashews.
Sweeteners	White sugar, brown sugar, maple syrup.	Agave, honey, fructose, xylitol, maltitol, mannitol, sorbitol.
